# Metallomic Aspects of Stroke and Recovery: ICP-MS Study with Chemometric Analysis

**DOI:** 10.3390/molecules30244672

**Published:** 2025-12-05

**Authors:** Bartłomiej Rospond, Aleksander Matusiak, Elżbieta U. Stolarczyk, Joanna Piotrowska, Bartosz Pomierny, Weronika Krzyżanowska, Przemysław W. Szafrański, Przemysław Dorożyński

**Affiliations:** 1 Department of Inorganic Chemistry and Pharmaceutical Analytics, Faculty of Pharmacy, Jagiellonian University Medical College, Medyczna 9 Street, 30-688 Kraków, Poland; bartlomiej.rospond@uj.edu.pl (B.R.); joanna.piotrowska@uj.edu.pl (J.P.); przemyslaw.dorozynski@uj.edu.pl (P.D.); 2 Spectrometric Methods Department, National Medicines Institute, 30/34 Chełmska Str., 00-725 Warsaw, Poland; e.stolarczyk@nil.gov.pl; 3 Center for the Development of Therapies for Civilization and Age-Related Diseases, Bioimaging Laboratory, Medyczna 9 Street, 30-688 Kraków, Poland; bartosz.pomierny@uj.edu.pl; 4 Department of Toxicological Biochemistry, Faculty of Pharmacy, Jagiellonian University Medical College, Medyczna 9 Street, 30-688 Kraków, Poland; weronika.krzyzanowska@uj.edu.pl; 5 Department of Organic Chemistry, Faculty of Pharmacy, Jagiellonian University Medical College, Medyczna 9 Street, 30-688 Kraków, Poland; p.szafranski@uj.edu.pl

**Keywords:** brain ischemia, inductively coupled plasma–mass spectrometry (ICP-MS), middle cerebral artery occlusion (MCAO), ischemic stroke, chemometric analysis, bioelements imbalance

## Abstract

Stroke remains a leading cause of death and disability worldwide, yet the contribution of elemental imbalance to its pathogenesis is not fully understood. Experimental evidence suggests that disturbances in the concentrations of essential and toxic elements contribute to neuronal injury through excitotoxicity, oxidative stress, and inflammation. In this study, we examined regional concentration in 15 elements (Na, K, Ca, Mg, P, Fe, Zn, Cu, Mn, Se, Cr, V, Pb, Al, B) in the subacute phase of ischemic stroke using the middle cerebral artery occlusion (MCAO) rat model. Male Sprague–Dawley rats underwent MCAO or sham surgery, after which the contralateral cortex, dorsal striatum, and hippocampus were collected seven days post-surgery. Elemental concentrations were determined by inductively coupled plasma-mass spectrometry (ICP-MS) and analyzed by Student’s *t*-test, cluster analysis, and principal component analysis (PCA). The *t*-test revealed widespread changes in Ca, while Na was least affected. PCA identified three principal components that explained 81.63% of the variance, with Mn, Zn, Se, K, Mg, Fe, and P contributing most strongly. Cluster analysis distinguished MCAO from sham groups and revealed region-specific responses. Our findings demonstrate long-lasting, region-dependent elemental imbalance after stroke, suggesting a valuable role of elemental profiling. Future investigations should aim to identify elements whose concentrations exhibit alterations not only within central nervous system regions but also in peripheral compartments, such as blood serum, as these changes may hold significant diagnostic and prognostic value.

## 1. Introduction

According to the Global Burden of Disease Study 2021, stroke is the second leading cause of death among non-communicable diseases, accounting for approximately 7 million fatalities worldwide annually. With over 160 million disability-adjusted life-years (DALYs) lost annually, it also ranks as the third leading cause of both death and disability globally [1]. In the United States, stroke ranks as the fifth leading cause of death, while in Europe, it is the primary cause of disability [2,3]. Therefore, there is an urgent, in-depth, ongoing research effort to better understand the disturbances in elemental homeostasis underlying stroke development. Middle cerebral artery occlusion (MCAO) is one of the most widely used animal models of stroke, reproducing both the ischemic core and the surrounding penumbra in the brains of rats and mice [4,5]. A key advantage of this model is the ability to precisely control the reperfusion duration by removing the occluding filament, enabling either transient or permanent ischemia, making it particularly suitable for studies on permanent ischemic injury [6,7]. Brain ischemia can alter the distribution of elements involved in cellular depolarization, such as Na, K, and Ca. Clinical evidence indicates that elevated urinary cadmium—derived mainly from smoking and dietary sources—as well as increased serum copper levels, may serve as an independent risk factor for ischemic stroke [8,9]. Cadmium shares chemical similarity with zinc and can interfere with the functions of zinc-containing proteins, particularly under zinc deficiency [10,11]. Moreover, increased concentrations of copper ions and copper-related proteins observed in rats subjected to an experimental stroke procedure have led to a new form of copper-induced cell death, termed cuproptosis [12,13,14]. In our previous study, we demonstrated that 24 h after stroke onset, the distribution of Fe, Mg, and Zn differs between ischemic and well-perfused brain regions [15]. Other reports indicate that an imbalance in elements such as Ca, K, and P may persist for up to one week after brain injury [16]. In the present study, we aimed to investigate a broader range of elements (15 in total) at a later time point, focusing on the subacute phase (7 days post-stroke). Our findings suggest that such investigations may provide valuable insights and potentially expand the current understanding of stroke pathophysiology and diagnosis [17,18]. Here, using the MCAO stroke model, we analyze contralateral structures—the hippocampus (HIPP), dorsal striatum (CDS), and frontal cortex (CC)—to identify significant changes in selected element concentrations, after seven days of stroke induction. The subacute phase after ischemic stroke represents the critical time for future recovery abilities, when elemental imbalances may be sustained and impact secondary injury mechanisms, including oxidative stress, excitotoxicity, and neuroinflammation. Previous evidence documented region-specific changes in Fe, Mg, and Zn within 24 h after stroke. We investigate here a broader spectrum of elements at seven days after stroke in order to ascertain longer-lasting changes that may contribute to continued neuronal damage and active repair processes. This not only advances understanding of stroke pathophysiology but also identifies potential element changes relevant to prognosis and therapeutic intervention.

## 2. Results

### 2.1. The Concentration of Elements

**Figure 1:**
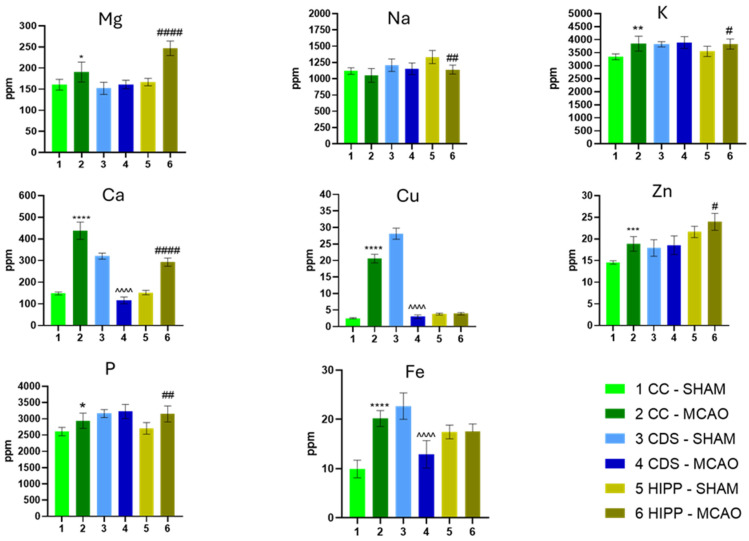
The concentrations of elements are expressed in ppm. The results are presented as the mean ± standard deviation (*n* = 6). Two-tailed Student *t*-test: * *p* < 0.05, ** *p* < 0.01, *** *p* < 0.001, **** *p* < 0.0001 vs. CC SHAM; ^^^^ *p* < 0.0001 vs. CDS SHAM; # *p* < 0.05, ## *p* < 0.01, #### *p* < 0.0001 vs. HIPP SHAM. Exact values are presented in the Supporting Information File.

**Figure 2:**
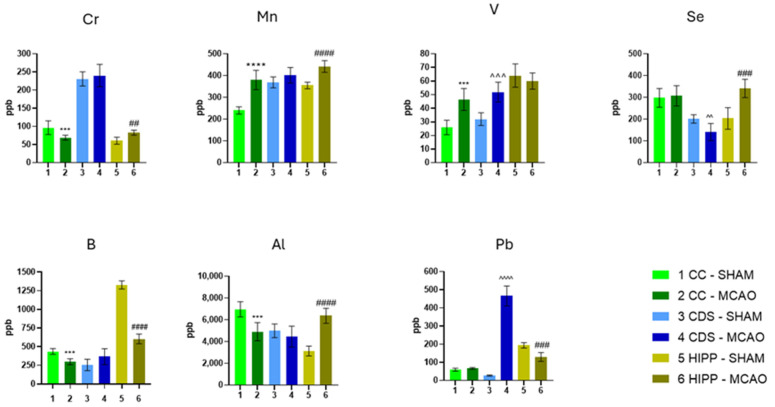
The concentrations of elements are expressed in ppb. The results are presented as the mean ± standard deviation (*n* = 6). Two-tailed Student *t*-test: *** *p* < 0.001, **** *p* < 0.0001 vs. CC SHAM; ^^ *p* < 0.01, ^^^ *p* < 0.001, ^^^^ *p* < 0.0001 vs. CDS SHAM; ## *p* < 0.01, ### *p* < 0.001, #### *p* < 0.0001 vs. HIPP SHAM. Exact values are presented in the Supporting Information File.

Figure 1 and Figure 2 present the concentrations of 15 chemical elements quantified in CC, DS, and HIPP across 6 experimental groups (*n* = 6 per group). The results are represented as mean ± standard deviation (SD), with MCAO vs. SHAM comparisons made per region using a two-tailed Student’s *t*-test (full statistics in the Electronic Supporting File, Table S1).

Across both figures, a region-specific pattern of elemental dysregulation emerged. Among all elements, calcium showed the broadest and most consistent alterations, with significant increases, especially in DS and HIPP, consistent with the central role of Ca^2+^-driven excitotoxicity in ischemic injury and its downstream cascades involving mitochondrial dysfunction and protease activation [19]. In contrast, sodium (Na) varied least, with significant change limited to the hippocampus.

Comparisons between CC and HIPP revealed differences in concentrations of magnesium (Mg), potassium (K), phosphorus (P), zinc (Zn), chromium (Cr), manganese (Mn), boron (B), and aluminum (Al). The largest increases appeared in HIPP and DS for Mg, Zn, K, P, and Al. Functionally, Mg and P are pivotal for adenosine 5′-triphosphate (ATP) dependent metabolism; Zn acts as a synaptic neuromodulator but contributes to oxidative/inflammatory injury when dysregulated in ischemia [20].

For copper (Cu), iron (Fe), and vanadium (V) levels in CC and DS, they diverged: increased in CC but decreased in DS. Given that Fe and Cu are redox-active, their regional accumulation can amplify reactive oxygen species (ROS) generation and lipid peroxidation. Links to iron-dependent damage and ferroptotic pathways are increasingly recognized in stroke [21,22].

Lead (Pb) and selenium (Se) showed reciprocal shifts between DS and HIPP (Pb↑/Se↓ in DS; Pb↓/Se↑ in HIPP). Pb is a well-established neurotoxicant associated with oxidative stress and synaptic dysfunction, whereas Se supports selenoprotein-mediated antioxidant defenses (e.g., GPX/GPX4). The opposite trends suggest a region-dependent balance between toxicant burden and endogenous antioxidant capacity [23,24].

Additional changes in Al, Cr, and Mn were evident, with the strongest increases in DS. Both Al and Mn have documented neurotoxic potential at elevated levels—Al via mitochondrial/oxidative impairments and Mn via nigrostriatal vulnerability and oxidative/inflammatory pathways, supporting their contribution to post-ischemic stress [25,26].

Overall, MCAO elicited heterogeneous, element-specific, and regionally distinct metal dyshomeostasis. Ca stood out as the most consistently altered element (aligning with excitotoxic signaling), Na remained least affected, and trace metals (Cu, Fe, V, Pb, Se) showed directionally opposite patterns across CC, DS, and HIPP, underscoring divergent vulnerabilities of white vs. gray-matter–dominant regions and the interplay of ionic imbalance and oxidative stress in ischemic neuropathology [19,21].

### 2.2. Chemometric Analysis: Cluster Analysis (CA) and Principal Component Analysis (PCA)

**Figure 3:**
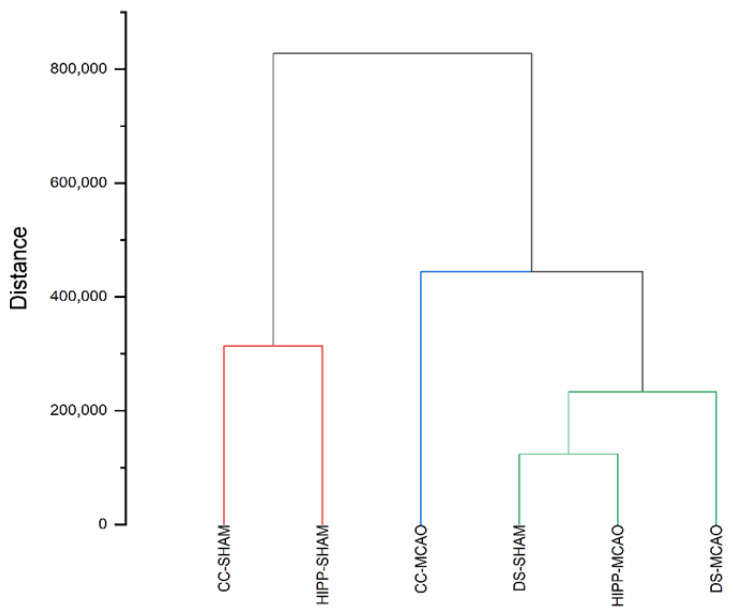
The cluster analysis (The Euclidean distance square, Furthest Neighbor algorithm) for three analyzed structures: Cortex, Dorsal Striatum, and Hippocampus. Groups are designated as follows: CC-SHAM, CC-MCAO, CDS-SHAM, CDS-MCAO, HIPP-SHAM, HIPP-MCAO.

The cluster analysis (CA) classified objects into groups (clusters) according to similar variability. The graphical representation of this analysis is the dendrogram, which is presented in Figure 3.

In our study, cluster analysis consists of three clusters: 1: CC-SHAM, HIPP-SHAM (red color); 2: CC-MCAO (blue color), 3: DS-SHAM, HIPP-MCAO, DS-MCAO (green color). The Euclidean metric was used to measure distance between points, and the farthest neighbor method calculated the distance between clusters. The *x*-axis represents the analyzed groups, and the *y*-axis shows the distances between them. As for the results, we can distinguish three main clusters described above. We can observe that SHAM animals tend to cluster together (CC-SHAM and HIPP-SHAM) and are in the same subgroup. MCAO animals/categories (with the procedure) form a second, larger cluster (DS + HIPP), although CC-MCAO behaves somewhat differently (more isolated). This suggests that the procedure (MCAO) changes the profile of the measured variables, but to different degrees depending on the region (CC, HIPP, DS). The clustering of DS-SHAM with MCAO groups strongly suggests that the basal elemental profile of the dorsal striatum mimics the changes seen in the ischemic group. This is likely rooted in the DS’s specific anatomy and high metabolic demands, which predispose it to stress-like characteristics even under baseline conditions. The striatum is located deep within the brain and is supplied by the lateral lenticulostriate arteries, which are terminal branches of the middle cerebral artery (MCA). Unlike the cerebral cortex (CC), the striatum has a relatively poor collateral blood supply in contrast to the highly vascularized cortex or the hippocampus. This makes the DS extremely vulnerable to the core ischemic injury following MCAO. The striatum is a highly dense and metabolically active structure, rich in GABAergic and cholinergic neurons and NMDA receptors, which makes it sensitive to excitotoxicity due to its high density during glutamate release.

Additionally, the separation of SHAM and MCAO clusters occurs at an even higher distance level (~800,000), highlighting clear differences between the control groups and those subjected to the procedure.

**Figure 4:**
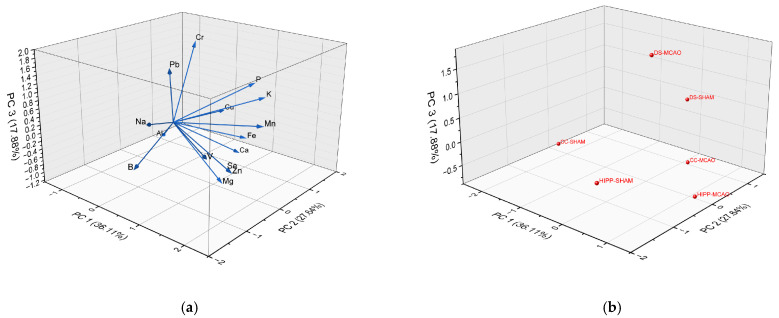
(**a**) The 3-dimensional presentation of the PCA analysis results; (**b**) The 3-dimensional presentation of the analysis structure in the PCA analysis.

PCA analysis was used to minimize the dataset to its most significant components and distinguish between more and less substantial information. Such analysis enabled us to reveal significant changes in element concentrations that affect the group under analysis. In the present paper, PCA analysis identified three principal components (principal component 1—PC1, principal component 2—PC2, and principal component 3—PC3), presented in Figure 4a,b and Table 1, which are linear combinations of input variables multiplied by the load values. The larger the charge value, the greater its influence on the principal component. In our experiment, those 3 Principal Components (PC1—36.11%, PC2—27.64%, PC3—17.88%) described 81.63% variability.

In PC1 component (36.11%), seven main essential elements: Mn, Zn, Se, K, Mg, Fe, P (micro- and macronutrients) have high positive loadings (>1.10). In PC2 (27, 64%), the contrast between calcium and copper profiles and boron and sodium is observed. In PC3 (17.88%), separation between toxic metals (Pb, Cr) from minerals (Mg, Ca, Al), Figure 4a.

Principal component analysis also revealed clear separation between different brain regions (CC, HIPP, CDS) and stroke and non-stroke groups. It could mean that each brain sample has a distinct structural and molecular profile, and stroke strongly affects the level of the investigated elements. The DS-MCAO and HIPP-MCAO groups are substantially separated from their respective controls (DS-SHAM and HIPP-SHAM), suggesting a robust and diverse response to stroke. The greatest distance is observed between CC-MCAO and CC-SHAM, suggesting that the cerebral cortex (CC) responded to the stroke differently or less strongly than the striatum (DS) and hippocampus (HIPP) (Figure 4b).

## 3. Discussion

The brain is highly vulnerable to ischemia because it relies primarily on blood glucose as its energy source. Inefficient transport of oxygen and glucose to the brain triggers a series of pathological events known as the ischemic cascade [27,28]. During the first 6 h after a stroke, the hyperacute phase of ischemia is defined mainly by excitotoxic K+ efflux from cells and Na+ influx into the cytosol. This leads to pronounced cellular depolarization, leading to cytotoxic edema [29]. Glutamate release activates glutamate receptors (NMDA, AMPA, and kainate receptors), increasing cell permeability to Na+, K+, and/or Ca^2+^ and initiation of intracellular calcium-dependent intracellular signaling pathways through enzymes, such as neuronal nitric oxide synthase (nNOS) and calcium/calmodulin-dependent protein kinase (CaMKs) [30,31].

As a consequence of these processes, cellular integrity is compromised, ionic imbalance develops, and free radicals are generated [32]. Over the following 6–48 h, the acute phase of ischemia occurs, which is characterized mainly by edema, oxidative stress, inflammation, and metabolic failure [33]. Elevated Ca^2+^ levels increase nitric oxide (NO) release, further activation of nNOS, and promote the production of reactive oxygen and nitrogen species, including superoxide (O_2_·), peroxynitrite (ONOO·), and hydroxyl radical (OH·). Among these, ONOO· is particularly harmful; generated mainly in mitochondria, it contributes to cellular membrane dysfunction, disruption of the blood–brain barrier (BBB), lipid peroxidation, DNA damage, mitochondrial respiratory chain impairment, and ATP depletion [34,35,36].

**Figure 5:**
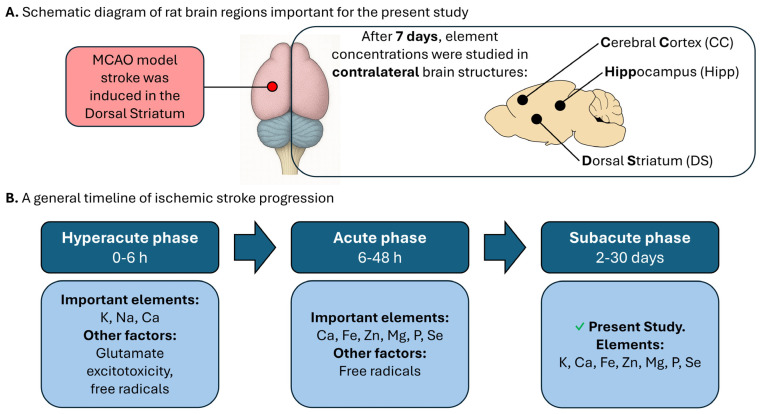
Experimental design and timeline of ischemic stroke progression in rat brain: (**A**) Schematic diagram of rat brain regions important for the present study. (**B**) Timeline of ischemic stroke phases, highlighting key elements and pathological factors. The present study focuses on the subacute phase, with the most important changes in K, Na, Ca, Fe, Zn, Mg, and Cu levels.

In the subsequent phase Activation of Inflammasomes and Relevant Modulators for the Treatment of Microglia-Mediated Neuroinflammation TNF-α), proapoptotic (ASK1, JNK, PTP), or anti-inflammatory (IL-4, IL-13, IGF-α) [36,37,38,39]. During the subacute phase (2–30 days after stroke), brain repair processes begin: swelling decreases, tissue regeneration occurs, and immune cells move and infiltrate the affected area [40,41]. The chronic phase involves long-term recovery and neuroadaptation. The stages of the ischemic cascade are summarized in Figure 5B.

In our study, element concentrations were measured 7 days after stroke induction (Figure 5A). This allowed us to assess changes occurring not in the acute, but in the subacute phase of stroke. According to the Student’s *t*-test results, the most significant alterations were observed in Ca levels; however, Principal Component Analysis classified Ca not within PC1, but in the PC2 cluster. This discrepancy reflects the specific characteristics of the subacute phase, during which glutamate excitotoxicity is no longer present. Principal Component Analysis (PC1) captures the overall pattern of variance across all 15 elements. PC1 is composed of a collective group of elements, e.g., Fe, Zn, and P, which are associated with later events like inflammation, oxidative stress, membrane breakdown, and ferroptosis. Ca is relegated to PC2 because its contribution to the overall pattern of change across all 15 elements is no longer dominant.

Furthermore, both PCA and Student’s *t*-test analyses revealed a minimal impact of Na (with the lowest statistical significance) and significant influence of K. When comparing these potassium results with our previous study [15], it can be observed that after 24 h of poststroke, K concentration had a much lower impact (0.94 PC3 after 24 h vs. 1.42 after 7 days). This suggests that the time of recovery influences K levels.

However, despite differences in recovery time (24 h vs. 7 days), Fe, Zn, and Mg consistently showed a similarly strong impact. Under physiological conditions, the BBB isolates the central nervous system from the peripheral vascular system and maintains stable brain iron levels. During cerebral ischemia, the BBB integrity is compromised, allowing iron-free forms (not bound to transferrin) to enter the brain [42]. Iron accumulation leads to lipid peroxidation (Fe is a key cofactor of lipoprotein lipase and cholesterol-7α-hydroxylase), initiating complex non-apoptotic pathways known as ferroptosis, which contribute to neuronal damage. Not only elevated but also reduced Fe levels can be harmful. Iron deficiency disrupts metabolic balance and promotes lipid accumulation, which readily crosses the BBB [42,43,44].

Animal studies of focal ischemia have shown that extracellular free zinc (ECF-Zn) increases slightly during the early phase of ischemia (30–75 min), followed by a threefold rise during the later phase (75–120 min) compared with sham controls. This effect is a result of the release of zinc from synaptic vesicles of ischemic neurons [45]. Elevated zinc levels during stroke may be detected not only in the brain but also in serum, as demonstrated by meta-analysis of clinical studies [46].

The magnesium levels in the CC MCAO and HIPP MCAO groups may reflect a compensatory response to increased calcium levels. Magnesium acts as a natural calcium antagonist by blocking vasoconstriction mediators, and calcium channels. It also enhances post-ischemic cerebral blood perfusion through vasodilation, thereby reducing ischemic injury [47,48].

Therefore, the neuroprotective role of magnesium has been investigated in both preclinical and clinical studies. In preclinical studies using a rat MCAO model, intravenous magnesium administration before ischemia and after reperfusion significantly reduces infarct volume. Magnesium serum concentrations above 2 mmol/L provided maximal neuroprotection and improved neurological recovery [49].

However, large clinical trials of FAST-MAG did not demonstrate a statistically significant reduction in mortality or overall stroke outcomes compared to placebo following magnesium intravenous administration [50].

Our study highlights the significant role of phosphorus (P) across all principal components (PC1, PC2, PC3). During ischemia, elevated phosphorus arises from the breakdown of membrane phospholipids, leading to the release of arachidonic acid and increased oxidative stress. Stroke also triggers the activation of phosphorus-containing mediators, such as lysophosphatidylcholine and sphingosine-1-phosphate, which contribute to phagocytosis [51,52]. MRI (Magnetic resonance spectroscopy) demonstrates a rapid decline in energy levels, particularly within the first minutes after stroke onset, which corresponds to early neuronal dysfunction [53]. Selenium is another element with a substantial impact on PC1. This element exerts multiple beneficial physiological effects; it stimulates glutathione peroxidase, reduces lipid peroxidation, and restores redox balance in brain tissue following ischemic injury [54,55]. It also modulates mitochondrial function and preserves mitochondrial integrity. In experimental stroke models, selenium pretreatment in hyperglycemic rats reduced the infarct volume [56]. Pretreatment with selenium organic form—selenocystamine or methylselenocysteine (0.5 mg/kg body weight for 5 days)—prevented the progression of neurological deficits in the MCAO mouse model and exhibited potential anti-ferroptotic properties [57]. Other studies have demonstrated its anti-inflammatory effects expressed as upregulation of Selenoprotein S (SelS) after 7 days of reperfusion [58,59].

In our experiment, Student’s *t*-test revealed a significant decrease in selenium levels in the stroke-affected region (DS), accompanied by an increase in the hippocampus, a structure not directly involved in stroke pathology, and no significant change in the cortex (penumbra region). These findings suggest a potential role for Se in stroke pathophysiology, as reduced Se levels in the ischemia-affected areas likely reflect the vulnerability of selenoproteins, the primary Se transporters, under ischemic conditions [60,61].

The last of the most influential elements in PC1 is manganese. The literature presents conflicting data regarding Mn levels in stroke. Some studies report decreased manganese concentrations in stroke patients [62], while others find no significant difference compared to control groups [63]. In our study, however, Mn levels were elevated in the stroke groups. Increased Mn concentration has been associated with enhanced BBB disruption and the promotion of oxidative stress [64]. The remaining analyzed elements, Cr, Mn, B, V, Pb, Cu, and Al, had a minor impact on PC1 but showed statistically significant differences between specific brain regions: Cr, Mn, and B in CC and CDS; V in CC and CDS; and Pb in CDS and HIPP. Cu, Al, Pb, and Mn are known to contribute to oxidative stress and inflammatory processes [65]. Clinical data indicate that the levels of certain toxic metals, such as Cd and Pb, are elevated in the blood during the stroke episodes [66]. However, their exact role in stroke pathophysiology remains to be fully elucidated and requires further investigation.

Another element of interest is copper. Clinical studies have shown that elevated Cu serum levels are associated with an increased risk of stroke [67,68]. Conversely, copper deficiency can impair antioxidant defenses and vascular function. Excess copper contributes to oxidative stress, vascular injury, and cell death through a recently described mechanism known as cuproptosis [69,70]. This novel form of cell death is distinct from apoptosis, necrosis, pyroptosis, and ferroptosis, and may play a role in stroke pathogenesis, warranting further investigation [71,72].

In our experiment, Cu levels decreased in the stroke-affected structure, CDS, and increased in the non-stroke region, CC. In PCA, Cu did not contribute to PC1, but was associated with PC2. This result is challenging to interpret, as the ambivalent values may indicate that Cu is more involved in antioxidant defense mechanisms than in the development of stroke itself. Copper and iron exhibit similar trends in concentration: they increase in CC and decrease in the Dorsal Striatum of the stroke group. This difference is seven days after a stroke, reflecting spatially distinct pathological events occurring in different brain regions. In DS, Ischemic Core, a massive cell death (necrosis, ferroptosis, cuproptosis) leads to the washout or efflux of intracellular metals from the destroyed tissue. In CC, elevated Fe can indicate toxic accumulation, continuing the risk of ferroptosis and expanding injury into the penumbra. The increase in Cu may represent a compensatory neuroprotective response by cells attempting to boost antioxidant capacity (e.g., SOD) against spreading oxidative stress. This theory could be supported by the fact that during the Blood–brain barrier disruption, easier metal-carrying components penetrated from the blood into the surrounding brain tissue. However, since we did not analyze copper-related secondary messengers, we were unable to assess how Cu dynamics may be functionally transferred.

This study aimed to analyze the concentrations of a broader range of elements (15 in total) in relation to stroke timing and across different brain structures (DS, CC, HIPP). Our goal was to obtain metallomic profiles and explore potential explanations for the observed changes. The summary is presented in Table 2. The scope of this work focused on elemental distribution and did not extend to investigating downstream effects on enzymatic activity, secondary messengers, or other molecular pathways, including epigenetic mechanisms.

## 4. Materials and Methods

### 4.1. Reagents

Nitric acid (HNO_3_, 69%) and Hydrogen peroxide (H_2_O_2_, 30%), both of Suprapur quality, were purchased from Merck (Darmstadt, Germany). Multi-Element ICP Calibration Standard (10 µg/mL each of Al, Ca, Cr, Cu, Fe, Mg, Mn, P, K, Se, Na, V, Zn, B in 3% HNO_3_) was acquired from Inorganic Ventures (Christiansburg, VA, USA). Elemental standard solutions (1000 mg/L) of indium (In) and bismuth (Bi) in 2–3% HNO_3_ were also purchased from Merck (Darmstadt, Germany). Scandium (Sc) elemental standard solution (10,000 µg/mL) in 5% HNO_3_ was obtained from VHG Labs (Manchester, NH, USA). A tuning solution for ICP-MS (1 µg/mL of Ce, Co, Li, Mg, Tl, and Y in 2% HNO_3_) was obtained from Agilent Technologies (Santa Clara, CA, USA). First-class water with a conductivity of 0.05 µS/cm was produced from the HLP 20UV water system (Hydrolab, Straszyn, Poland).

### 4.2. Animals

**Figure 6:**
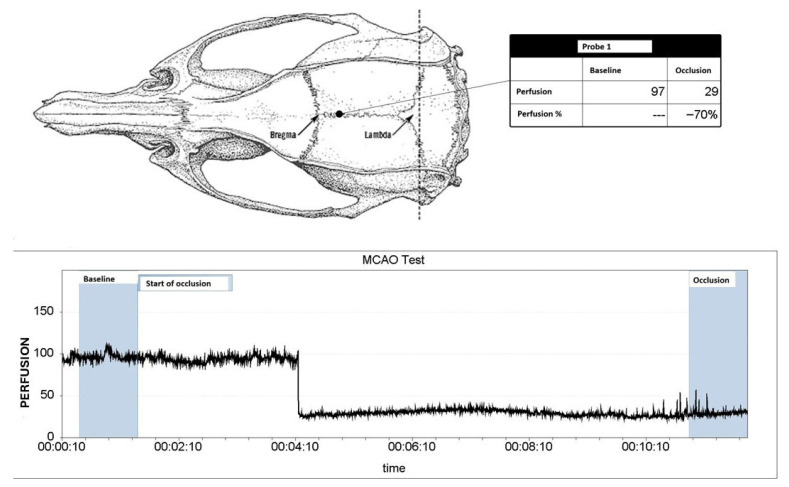
The report from the laser Doppler flowmetry illustrates a decrease in blood flow in the middle cerebral artery following occlusion.

**Figure 7:**
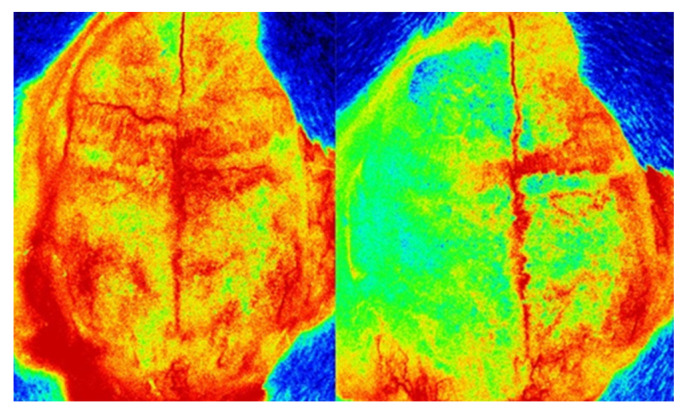
Blood flow imaging in the brain arteries obtained from LSCI before MCAO occlusion (**left**), and after MCAO occlusion (**right**).

**Figure 8:**
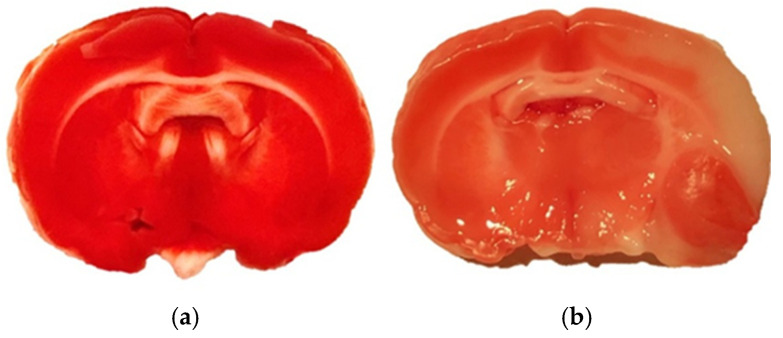
TTC staining (2,3,5-triphenyltetrazolium chloride) was used for detecting the viability of tissues: (**a**) living cells reduced colorless TTC to red formazan, (**b**) dead tissues remain colorless.

All experimental methods were carried out in accordance with the National Institutes of Health’s Guide for the Care and Use of Laboratory Animals and were authorized by Jagiellonian University’s First Local Ethical Committee in Krakow (permission numbers 11/2017 & 12/2017) [73]. The ARRIVE (Animal Research: Reporting of In Vivo Experiments) criteria, which include blinding researchers to the animals’ identities at every stage of the experiment, were followed in the reporting of all animal-based studies [74]. The electronic supporting Information contains detailed processes similar to those in Refs. [15,75]. Male Sprague Dawley rats (280–320 g, Charles River, *n* = 6 per group) were used for all experiments and kept at 22 ± 2 °C with free access to food and water on a regular day-night cycle. Rats were divided into the following groups at random: (1) SHAM (sham surgery), (2) MCAO (Middle Cerebral Artery Occlusion). Tissues were isolated 7 days after surgery. A stereoscopic microscope (Leica A60F, Leica, Wetzlar, Germany) was used for all surgical procedures, and a heating blanket (Homeothermic Blanket System; Harvard Apparatus, Holliston, MA, USA) was used to keep body temperature at a physiological level. A laser Doppler flowmetry equipment (PeriFlux System 5000, Perimed, Järfälla, Sweden) was used to detect arterial occlusion, and a reduction of 70% in blood flow in the MCA region (Figure 6) was deemed indicative of a successful artery occlusion. 5% isoflurane was used to induce anesthesia in the rats, and 2.5% to maintain it. This successful MCAO occlusion was also confirmed by images obtained from laser speckle contrast imaging (LSCI, PeriCam PSI, Perimed, Järfälla, Sweden Figure 7). Figure 8 shows an example of histopathological staining with TTC (2,3,5-triphenyltetrazolium chloride), confirming the formation of a necrotic area following MCAO.

All the ECA’s branches were coagulated following the exposure of the left external carotid artery (ECA), internal carotid artery (ICA), and common carotid artery (CCA). Microvascular clips were used to anchor the ECA, ICA, and CCA temporarily. The silicone-coated filament (Doccol, Sharon, MA, USA) was introduced into the ECA lumen and further advanced until it reached the MCA. Silk sutures were used to close the wound. For ninety minutes, the occlusion was maintained. The wound was then reopened, and the filament was removed to allow for blood reperfusion. Seven days after SHAM or MCAO operation, rats were sacrificed and selected contralateral brain structures were isolated: CC (Cortex), CDS (Dorsal Striatum), and HIPP (Hippocampus), and kept at −80 °C until further analysis. 

### 4.3. Sample Preparation

Whole obtained structures (CC, DS, HIPP) were weighted on an analytical balance with 0.00001 g accuracy (Mettler-Toledo AG, Greifensee, Switzerland) and digested in the Milestone ultraWAVE (Milestone Srl, Sorisole, Italy) single reaction chamber digestion system, with the program presented in Table 3. Samples were placed in quartz vials, then 4 mL of HNO_3_ and 1 mL of H_2_O_2_ were added. The starting loading pressure was 40 bar with nitrogen as a loading gas. After digestion, each sample was diluted with water to a final HNO_3_ concentration of about 5.5%.

Calibration working standards were prepared by diluting the multi-element ICP calibration standard with 5.5% HNO_3_ to match its concentration in the samples. An internal standard solution was prepared by mixing and diluting single-element calibration standards.

### 4.4. Instrumentation

An Agilent 7800 single-quadrupole ICP-MS with SPS 4 Autosampler (Agilent Technologies, Santa Clara, CA, USA) was used to measure element concentrations. The instrument was equipped with a MicroMist glass nebulizer, a Scott double-pass quartz spray chamber, a 2.5 mm ID quartz injector torch, a Ni-tipped copper sampling cone, a Ni skimmer cone, and an external internal standard delivery line for samples. The spectrometer was tuned daily with Tuning Solution. Operating conditions are presented in Table 4.

The concentration of elements was measured with and without He collision gas. ^11^B, ^24^Mg, ^31^P, ^39^K, ^44^Ca, ^51^V, ^52^Cr, ^55^Mn, ^56^Fe, ^63^Cu, ^66^Zn, ^78^Se, ^206^Pb, ^207^Pb and ^208^Pb were measured with He collision gas. ^23^Na and ^27^Al were measured without He collision gas. The internal standard was a mixture of ^45^Sc, ^115^In, and ^209^Bi at 500 ppb each. ^45^Sc was used as internal standard for ^11^B, ^23^Na, ^24^Mg, ^27^Al, ^31^P, ^39^K, ^44^Ca, ^51^V, ^52^Cr, ^55^Mn, ^56^Fe, ^115^In as internal standard for ^63^Cu, ^66^Zn, ^78^Se, and finally ^209^Bi as internal standard for ^206^Pb, ^207^Pb and ^208^Pb. The concentration range for all measured elements ranged from 1 μg/L to 1000 μg/L. Analysis details, including the limits of detection (LOD), limits of quantification (LOQ), and the correlation coefficient (R), are presented in Table 5. LOD concentrations were calculated based on the SD of a response measured in a blank solution and the calibration curve slope. LOQs were calculated as 3 times the LOD.

### 4.5. Data Analysis

The significant difference between (SHAM vs. MCAO) was investigated using a two-tailed Student’s *t*-test. The resulting element levels were presented as the mean ± standard deviation (SD). A significance level of *p* < 0.05 was used to determine statistical significance between groups. Data analaysis was performed using GraphPad/Prism 8 software (GraphPad Software, Boston, MA, USA) and the Origin 2025b (OriginLab Corporation, Northampton, MA, USA) program for chemometric analysis (PCA—Principal Component Analysis and CA—Cluster Analysis with the Furthest Neighbor Method to determine distances between clusters). The results were standardized before applying the chemometric method.

## 5. Conclusions

This study investigated the concentrations of 15 elements in 3 distinct brain regions: cerebral cortex (CC), dorsal striatum (DS), and hippocampus (HIPP) during the subacute phase of ischemic stroke (7 days post-stroke). Elemental concentrations were analyzed using Student’s *t*-test and advanced chemometric methods, including cluster analysis (CA) and principal component analysis (PCA).

The analysis revealed clear region-specific patterns of metal dyshomeostasis following MCAO. Among all elements, calcium (Ca) exhibited the most consistent and pronounced alterations, highlighting its central role in excitotoxic signaling and downstream ischemic cascades. In contrast, sodium (Na) showed minimal variability. Seven elements—Mn, Zn, Se, K, Mg, Fe, and P—had the most decisive influence on PC1, which accounted for 36.11% of the total variance. These elements represent key micro- and macronutrients involved in ionic homeostasis, oxidative stress regulation, mitochondrial function, and metabolic activity.

We observed opposing trends in several redox-active elements, including Cu, Fe, Pb, and Se, across different brain regions, indicating divergent regional vulnerability. Trace metals with neurotoxic potential, such as Pb, Al, and Mn, showed significant increases, particularly in DS, suggesting their contribution to post-ischemic oxidative and inflammatory stress.

Cluster analysis revealed distinct separation between SHAM and MCAO groups, with DS and HIPP showing stronger responses than CC. PCA further demonstrated region-dependent molecular signatures, reflecting the heterogeneity of ischemic injury across brain structures.

The findings indicate that ischemic stroke induces element-specific and regionally distinct metal imbalances, with Ca as a key driver of excitotoxic damage and trace metals contributing to oxidative stress. The metallomic profile identified in this study highlights the complex interplay between essential and toxic elements in post-ischemic brain tissue and underscores their potential mechanistic roles in stroke pathology. When compared with the findings of Ali et al. (2018) [76], despite differences between our MCAO model and their photothrombotic paradigm, several parallels emerge, particularly the regional accumulation of Ca, Zn, and Fe and the pronounced metal dyshomeostasis observed following ischemia. As in [76], which used PCA and cluster analysis for IR data, we found that chemometric analysis can provide additional clues for interpreting metal concentration-related data.

Our study analyzed a wide range of elements (15 in total), providing a comprehensive “metallomic profile” rather than focusing on a few elements, which are more valuable and reliable for identifying elemental changes. The design of the experiment (7 days after stroke appearance) allowed the researchers to assess: changes specific to the subacute recovery/repair phase, which is distinct from the immediate acute phase, differences between the core ischemic region (DS), a non-stroke region (HIPP), and potentially the penumbra region (Cortex). In this article, the statistical methods chemometric analysis (PCA) and Integration of Statistical Methods: the use of Principal Component Analysis (PCA) and Student’s *t*-test provided a robust analytical framework, helping to classify elements into clusters based on their overall impact and explore discrepancies (e.g., for Ca).

This study provides a new context for previously observed trends in elemental imbalance by comparing element concentrations after 7 days versus 24 h after the stroke operation. Furthermore, it suggests novel involvement for elements such as P, Se, and Mn in the subacute phase, opening new avenues for research (e.g., the potential anti-ferroptotic role of Se). The discussion relates the observed elemental changes to relevant stroke pathology and recovery mechanisms, including a detailed discussion about the element’s role. It could have diagnostic implications—whether supplementation of a given element can be beneficial or not.

This study did not investigate downstream effects at the level of enzymatic activity, secondary messengers, molecular pathways, or epigenetic mechanisms. This limitation means the observed elemental changes cannot be “functionally transferred” directly. Moreover, due to the ICP-MS analytical method used in this experiment, bound or free states were not determined in this work (e.g., Fe not bound to transferrin).

Future studies should focus on linking elemental dyshomeostasis to molecular and cellular pathways, including enzymatic activity, secondary messengers, and epigenetic regulation, to better understand the mechanistic role of trace elements in stroke progression and recovery.

## Figures and Tables

**Table 1:** Characteristics of PC1, PC2, PC3 components.

Element	PC1 36.11%	PC2 27.64%	PC3 17.88%
B	0.51	−1.61	−0.35
Na	0.39	−1.15	0.49
Mg	1.20	0.02	−1.17
K	1.42	1.03	0.70
Ca	0.64	1.32	−1.13
V	1.60	−0.95	0.02
Cr	−0.11	0.80	1.81
Fe	1.10	0.92	−0.41
Cu	0.12	1.46	−0.16
Zn	1.80	−0.49	−0.44
Se	1.80	−0.49	−0.44
Pb	0.49	−0.65	1.68
Mn	1.85	0.38	0.34
Al	−0.82	0.58	−0.86
P	1.19	1.01	1.01

**Table 2:** Summary of observed element concentration changes in contralateral brain structures 7 days after MCAO stroke procedure. Arrow up (↑)—statistically significant growth, arrow down (↓)—statistically significant decrease, wavy line (~)—no statistically significant change. Double arrows indicate a large magnitude of effect (increase or decrease).

Category	Element	CC	DS.	HIPP	Functional Significance/Interpretation
I. Macroelements	Ca	↑↑	↓↓	↑	Consistent with Ca^2+^-driven excitotoxicity, mitochondrial dysfunction, and protease activation in ischemic injury.
	Na	~	~	↓	Varied least among all elements; suggests Na imbalance is less dominant in the subacute phase.
	K	↑	~	↑	Linked to ionic imbalance and key for ATP-dependent metabolism and cell function.
	Mg	↑	~	↑↑	Pivotal for ATP-dependent metabolism; acts as a natural Ca^2+^ antagonist.
	P	↑	~	↑	Pivotal for ATP-dependent metabolism and structural membrane integrity.
II Antioxidants	Fe	↑↑	↓↓	~	Redox-active; Fe accumulation ↑ ROS and lipid peroxidation; linked to ferroptotic pathways in stroke.
	Cu	↑↑	↓↓	~	Redox-active; accumulation ↑ ROS generation. Opposite trends underscore divergent regional responses (defense vs. destruction).
	Zn	↑	~	↑	Synaptic neuromodulator; dysregulation contributes to oxidative/inflammatory injury.
	Se	~	↓	↑↑	Supports selenoprotein-mediated antioxidant defenses (GPX). Suggests region-dependent vulnerability/capacity.
III Toxicants	Pb	~	↑↑	↓	Well-established neurotoxicant associated with oxidative stress and synaptic dysfunction.
	V	↑↑	↑↑	~	Trace metal showing opposite trends between cortex and striatum.
IV. Trace & Neurotoxic	Mn	↑↑	~	↑	Documented neurotoxic potential at high levels via oxidative/inflammatory pathways.
	Al.	↓	~	↑↑	Documented neurotoxic potential via mitochondrial and oxidative impairments.
	Cr	↓	~	↑	Trace metal showing regional differences.
	B	↓	~	↓↓	Trace metal showing regional differences.

**Table 3:** Sample digestion parameters.

Step	Time (Min)	Power (W)	Internal Temperature (°C)	External Temperature (°C)	Pressure (Bar)
1	10:00	800	110	60	80
2	10:00	1000	180	60	80
3	10:00	1500	240	60	120
4	10:00	1500	240	60	120

**Table 4:** ICP-MS operating conditions.

ICP-MS Parameter	Operating Mode
RF power (W)	1550
Sample depth (mm)	10.0
Nebulizer gas flow (L/min)	0.99
Plasma gas flow (L/min)	15
Auxiliary gas flow (L/min)	0.9
Cell gas flow in He mode (mL/min)	4.3 mL/min
Lens Tune	Autotune

**Table 5:** Basic validation parameters for ICP-MS. R—correlation coefficient, LOD—Limit of Detection, LOQ—Limit of Quantification.

Element	R	LOD [ppb]	LOQ [ppb]
^11^B	1.0000	0.34	1.0
^23^Na	0.9994	0.27	0.82
^24^Mg	0.9997	0.14	0.42
^27^Al	0.9997	0.039	0.12
^31^P	0.9996	34	101
^39^K	0.9998	17	50
^44^Ca	0.9980	0.62	1.9
^51^V	1.0000	0.0054	0.016
^52^Cr	1.0000	0.029	0.087
^55^Mn	1.0000	0.012	0.037
^56^Fe	1.0000	0.42	1.3
^63^Cu	1.0000	0.037	0.11
^66^Zn	0.9999	0.21	0.63
^78^Se	1.0000	0.17	0.52
Pb *	1.0000	0.010	0.030

* as sum of ^206^Pb, ^207^Pb and ^208^Pb.

## Data Availability

The original contributions presented in this study are included in this article. Further inquiries can be directed to the corresponding author.

## Supplementary Materials

The following supporting information can be downloaded at: https://www.mdpi.com/article/10.3390/molecules30244672/s1, Table S1. The range of concentration of elements and the observed statistical significances.

## Abbreviations

The following abbreviations are used in this manuscript:

ATP	Adenosine 5′-triphosphate
BBB	Blood–brain barrier
CA	Cluster analysis
CC	Frontal cortex
CDS	Dorsal striatum
DALYs	Disability-adjusted life-years
HIPP	Hippocampus
ICP-MS	Inductively coupled plasma-mass spectrometry
LOD	Limit of detection
LOQ	Limit of quantification
MCAO	Middle cerebral artery occlusion
nNOS	Neuronal nitric oxide synthase
PCA	Principal component analysis
ROS	Reactive oxygen species
SD	Standard deviation

## References

[1] Feigin V.L., Brainin M., Norrving B., Martins S.O., Pandian J., Lindsay P., Grupper M.F., Rautalin I. (2025). World Stroke Organization: Global Stroke Fact Sheet 2025. Int. J. Stroke.

[2] Herpich F., Rincon F. (2020). Management of Acute Ischemic Stroke. Crit. Care Med..

[3] Soto A., Guillén-Grima F., Morales G., Muñoz S., Aguinaga-Ontoso I., Fuentes-Aspe R. (2022). Prevalence and Incidence of Ictus in Europe: Systematic Review and Meta-Analysis. An. Sist. Sanit. Navar..

[4] Sheng H., Dang L., Li X., Yang Z., Yang W. (2023). A Modified Transcranial Middle Cerebral Artery Occlusion Model to Study Stroke Outcomes in Aged Mice. J. Vis. Exp..

[5] Narayan S.K., Cherian S.G., Phaniti P.B., Chidambaram S.B., Vasanthi A.H.R., Arumugam M. (2021). Preclinical Animal Studies in Ischemic Stroke: Challenges and Some Solutions. Anim. Model. Exp. Med..

[6] MacRae I. (2011). Preclinical Stroke Research—Advantages and Disadvantages of the Most Common Rodent Models of Focal Ischaemia. Br. J. Pharmacol..

[7] Zeng L., Hu S., Zeng L., Chen R., Li H., Yu J., Yang H. (2023). Animal Models of Ischemic Stroke with Different Forms of Middle Cerebral Artery Occlusion. Brain Sci..

[8] Chen C., Xun P., Tsinovoi C., McClure L.A., Brockman J., MacDonald L., Cushman M., Cai J., Kamendulis L., Mackey J. (2018). Urinary Cadmium Concentration and the Risk of Ischemic Stroke. Neurology.

[9] Poulsen A.H., Sears C.G., Harrington J., Howe C.J., James K.A., Roswall N., Overvad K., Tjønneland A., Wellenius G.A., Meliker J. (2021). Urinary Cadmium and Stroke—A Case-Cohort Study in Danish Never-Smokers. Environ. Res..

[10] Tang L., Qiu R., Tang Y., Wang S. (2014). Cadmium-Zinc Exchange and Their Binary Relationship in the Structure of Zn-Related Proteins: A Mini Review. Metallomics.

[11] Zhang M., Li W., Wang Y., Wang T., Ma M., Tian C. (2020). Association Between the Change of Serum Copper and Ischemic Stroke: A Systematic Review and Meta-Analysis. J. Mol. Neurosci..

[12] Peng G., Huang Y., Xie G., Tang J. (2024). Exploring Copper’s Role in Stroke: Progress and Treatment Approaches. Front. Pharmacol..

[13] Kahlson M.A., Dixon S.J. (2022). Copper-Induced Cell Death. Science.

[14] Guo Q., Ma M., Yu H., Han Y., Zhang D. (2023). Dexmedetomidine Enables Copper Homeostasis in Cerebral Ischemia/Reperfusion via Ferredoxin 1. Ann. Med..

[15] Rospond B., Krakowska A., Piotrowska J., Pomierny B., Krzyżanowska W., Szewczyk B., Szafrański P., Dorożynski P., Paczosa-Bator B. (2025). Multidimensional Analysis of Selected Bioelements in Rat’s Brain Subjected to Stroke Procedure and Treatment with H2S Donor AP-39. J. Trace Elem. Med. Biol..

[16] Siegele R., Howell N.R., Callaghan P.D., Pastuovic Z. (2013). Investigation of Elemental Changes in Brain Tissues Following Excitotoxic Injury. Nucl. Instrum. Methods Phys. Res. B.

[17] Pushie M.J., Sylvain N.J., Hou H., Pendleton N., Wang R., Zimmermann L., Pally M., Cayabyab F.S., Peeling L., Kelly M.E. (2024). X-Ray Fluorescence Mapping of Brain Tissue Reveals the Profound Extent of Trace Element Dysregulation in Stroke Pathophysiology. Metallomics.

[18] Pushie M.J., Sylvain N.J., Hou H., George D., Kelly M.E. (2024). Ion Dyshomeostasis in the Early Hyperacute Phase after a Temporary Large-Vessel Occlusion Stroke. ACS Chem. Neurosci..

[19] Szydlowska K., Tymianski M. (2010). Calcium, Ischemia and Excitotoxicity. Cell Calcium.

[20] Sensi S.L., Paoletti P., Bush A.I., Sekler I. (2009). Zinc in the Physiology and Pathology of the CNS. Nat. Rev. Neurosci..

[21] Tuo Q.Z., Lei P., Jackman K.A., Li X.L., Xiong H., Li X.L., Liuyang Z.Y., Roisman L., Zhang S.T., Ayton S. (2017). Tau-Mediated Iron Export Prevents Ferroptotic Damage after Ischemic Stroke. Mol. Psychiatry.

[22] Dixon S.J., Stockwell B.R. (2014). The Role of Iron and Reactive Oxygen Species in Cell Death. Nat. Chem. Biol..

[23] Flora G., Gupta D., Tiwari A. (2012). Toxicity of Lead: A Review with Recent Updates. Interdiscip. Toxicol..

[24] Shayganfard M. (2022). Are Essential Trace Elements Effective in Modulation of Mental Disorders? Update and Perspectives. Biol. Trace Elem. Res..

[25] Kawahara M., Kato-Negishi M. (2011). Link between Aluminum and the Pathogenesis of Alzheimer’s Disease: The Integration of the Aluminum and Amyloid Cascade Hypotheses. Int. J. Alzheimers Dis..

[26] Guilarte T.R. (2010). Manganese and Parkinson’s Disease: A Critical Review and New Findings. Environ. Health Perspect..

[27] Rigual R., Fuentes B., Díez-Tejedor E. (2023). Management of Acute Ischemic Stroke. Med. Clin..

[28] Candelario-Jalil E. (2009). Injury and Repair Mechanisms in Ischemic Stroke: Considerations for the Development of Novel Neurotherapeutics. Curr. Opin. Investig. Drugs.

[29] Przykaza Ł. (2021). Understanding the Connection Between Common Stroke Comorbidities, Their Associated Inflammation, and the Course of the Cerebral Ischemia/Reperfusion Cascade. Front. Immunol..

[30] Mehta S.L., Manhas N., Raghubir R. (2007). Molecular Targets in Cerebral Ischemia for Developing Novel Therapeutics. Brain Res. Rev..

[31] Rahimpour S., Meadows E., Hollander J.M., Karelina K., Brown C.M. (2025). Assessment of Phase-Dependent Alterations in Cortical Glycolytic and Mitochondrial Metabolism Following Ischemic Stroke. ASN Neuro.

[32] Giffard R.G., Swanson R.A. (2005). Ischemia-Induced Programmed Cell Death in Astrocytes. Glia.

[33] Xu A., Zhang H., Zhang Y., Wu J., Huang Z. (2025). Ischemic Stroke and Intervention Strategies Based on the Timeline of Stroke Progression: Review and Prospects. Acta Pharm. Sin. B.

[34] Martynov M.Y., Zhuravleva M.V., Vasyukova N.S., Kuznetsova E.V., Kameneva T.R. (2023). Oxidative Stress in the Pathogenesis of Stroke and Its Correction. Zhurnal Nevrologii i Psihiatrii imeni S.S. Korsakova.

[35] Parvez S., Kaushik M., Ali M., Alam M.M., Ali J., Tabassum H., Kaushik P. (2022). Dodging Blood Brain Barrier with “Nano” Warriors: Novel Strategy against Ischemic Stroke. Theranostics.

[36] Zhang R., Xu M., Wang Y., Xie F., Zhang G., Qin X. (2017). Nrf2-a Promising Therapeutic Target for Defensing Against Oxidative Stress in Stroke. Mol. Neurobiol..

[37] Zeng Z.J., Lin X., Yang L., Li Y., Gao W. (2024). Activation of Inflammasomes and Relevant Modulators for the Treatment of Microglia-Mediated Neuroinflammation in Ischemic Stroke. Mol. Neurobiol..

[38] Garcia-Bonilla L., Shahanoor Z., Sciortino R., Nazarzoda O., Racchumi G., Iadecola C., Anrather J. (2024). Analysis of Brain and Blood Single-Cell Transcriptomics in Acute and Subacute Phases after Experimental Stroke. Nat. Immunol..

[39] Weber R.Z., Achón Buil B., Rentsch N.H., Bosworth A., Zhang M., Kisler K., Tackenberg C., Rust R. (2025). A Molecular Brain Atlas Reveals Cellular Shifts during the Repair Phase of Stroke. J. Neuroinflammation.

[40] Enzmann G., Kargaran S., Engelhardt B. (2018). Ischemia-Reperfusion Injury in Stroke: Impact of the Brain Barriers and Brain Immune Privilege on Neutrophil Function. Ther. Adv. Neurol. Disord..

[41] Nesto R.W., Kowalchuk G.J. (1987). The Ischemic Cascade: Temporal Sequence of Hemodynamic, Electrocardiographic and Symptomatic Expressions of Ischemia. Am. J. Cardiol..

[42] Guo J., Tuo Q., Lei P. (2023). Iron, Ferroptosis, and Ischemic Stroke. J. Neurochem..

[43] Dixon S.J., Pratt D.A. (2023). Ferroptosis: A Flexible Constellation of Related Biochemical Mechanisms. Mol. Cell.

[44] Pifferi F., Laurent B., Plourde M. (2021). Lipid Transport and Metabolism at the Blood-Brain Interface: Implications in Health and Disease. Front. Physiol..

[45] Qi Z., Zhou X., Dong W., Timmins G.S., Pan R., Shi W., Yuan S., Zhao Y., Ji X., Liu K.J. (2023). Neuronal Zinc Transporter ZnT3 Modulates Cerebral Ischemia-Induced Blood-Brain Barrier Disruption. Aging Dis..

[46] Huang M., Zhu L., Chen Y., Jin Y., Fang Z., Yao Y. (2022). Serum/Plasma Zinc Is Apparently Increased in Ischemic Stroke: A Meta-Analysis. Biol. Trace Elem. Res..

[47] Xu R., Wang L., Sun L., Dong J. (2021). Neuroprotective Effect of Magnesium Supplementation on Cerebral Ischemic Diseases. Life Sci..

[48] Lin J.Y., Chung S.Y., Lin M.C., Cheng F.C. (2002). Effects of Magnesium Sulfate on Energy Metabolites and Glutamate in the Cortex during Focal Cerebral Ischemia and Reperfusion in the Gerbil Monitored by a Dual-Probe Microdialysis Technique. Life Sci..

[49] Westermaier T., Zausinger S., Baethmann A., Schmid-Elsaesser R. (2005). Dose Finding Study of Intravenous Magnesium Sulphate in Transient Focal Cerebral Ischemia in Rats. Acta Neurochir..

[50] Fan S., Jang M., Kim-Tenser M., Shkirkova K., Liebeskind D.S., Starkman S., Villablanca J.P., Hamilton S., Naidech A., Saver J.L. (2023). Effect of Magnesium on Deterioration and Symptomatic Hemorrhagic Transformation in Cerebral Ischemia: An Ancillary Analysis of the FAST-MAG Trial. Cerebrovasc. Dis..

[51] Cheng J., Wang W., Xia Y., Li Y., Jia J., Xiao G. (2023). Regulators of Phagocytosis as Pharmacologic Targets for Stroke Treatment. Front. Pharmacol..

[52] Adibhatla R.M., Hatcher J.F. (2006). Phospholipase A2, Reactive Oxygen Species, and Lipid Peroxidation in Cerebral Ischemia. Free Radic. Biol. Med..

[53] Taylor J.M., Zhu X.H., Zhang Y., Chen W. (2015). Dynamic Correlations between Hemodynamic, Metabolic, and Neuronal Responses to Acute Whole-Brain Ischemia. NMR Biomed..

[54] Ansari M.A., Ahmad A.S., Ahmad M., Salim S., Yousuf S., Ishrat T., Islam F. (2004). Selenium Protects Cerebral Ischemia in Rat Brain Mitochondria. Biol. Trace Elem. Res..

[55] Özbal S., Erbil G., Koçdor H., Tuǧyan K., Pekçetin Ç., Özoǧul C. (2008). The Effects of Selenium against Cerebral Ischemia-Reperfusion Injury in Rats. Neurosci. Lett..

[56] Yang L., Ma Y.M., Shen X.L., Fan Y.C., Zhang J.Z., Li P.A., Jing L. (2020). The Involvement of Mitochondrial Biogenesis in Selenium Reduced Hyperglycemia-Aggravated Cerebral Ischemia Injury. Neurochem. Res..

[57] Tuo Q.Z., Masaldan S., Southon A., Mawal C., Ayton S., Bush A.I., Lei P., Belaidi A.A. (2021). Characterization of Selenium Compounds for Anti-Ferroptotic Activity in Neuronal Cells and After Cerebral Ischemia–Reperfusion Injury. Neurotherapeutics.

[58] Liu L.X., Zhou X.Y., Li C.S., Liu L.Q., Huang S.Y., Zhou S.N. (2013). Selenoprotein S Expression in the Rat Brain Following Focal Cerebral Ischemia. Neurol. Sci..

[59] Fradejas N., Del Carmen Serrano-Pérez M., Tranque P., Calvo S. (2011). Selenoprotein S Expression in Reactive Astrocytes Following Brain Injury. Glia.

[60] Zhuo Z., Wang H., Zhang S., Bartlett P.F., Walker T.L., Hou S.T. (2023). Selenium Supplementation Provides Potent Neuroprotection Following Cerebral Ischemia in Mice. J. Cereb. Blood Flow. Metab..

[61] Turovsky E.A., Mal’tseva V.N., Sarimov R.M., Simakin A.V., Gudkov S.V., Plotnikov E.Y. (2022). Features of the Cytoprotective Effect of Selenium Nanoparticles on Primary Cortical Neurons and Astrocytes during Oxygen–Glucose Deprivation and Reoxygenation. Sci. Rep..

[62] Gönüllü H., Karadaş S., Milanlioğlu A., Gönüllü E., Katı C., Demir H. (2014). Levels of serum trace elements in ischemic stroke patients. J. Exp. Clin. Med..

[63] Jamebozorgi K., Kooshki A., Saljoughi M., Sanjari M., Ahmadi Z., Mosavi Mirzaei S.M. (2025). Cerebrovascular Accidents Association between Serum Trace Elements and Toxic Metals Level, a Case-Control Study. PLoS ONE.

[64] Oliveira-Paula G.H., Martins A.C., Ferrer B., Tinkov A.A., Skalny A.V., Aschner M. (2024). The Impact of Manganese on Vascular Endothelium. Toxicol. Res..

[65] Wen Y., Huang S., Zhang Y., Zhang H., Zhou L., Li D., Xie C., Lv Z., Guo Y., Ke Y. (2019). Associations of Multiple Plasma Metals with the Risk of Ischemic Stroke: A Case-Control Study. Environ. Int..

[66] Mirończuk A., Kapica-Topczewska K., Socha K., Soroczyńska J., Jamiołkowski J., Chorąży M., Czarnowska A., Mitrosz A., Kułakowska A., Kochanowicz J. (2023). Disturbed Ratios between Essential and Toxic Trace Elements as Potential Biomarkers of Acute Ischemic Stroke. Nutrients.

[67] Yang L., Chen X., Cheng H., Zhang L. (2022). Dietary Copper Intake and Risk of Stroke in Adults: A Case-Control Study Based on National Health and Nutrition Examination Survey 2013–2018. Nutrients.

[68] Bao Q.J., Zhao K., Guo Y., Wu X.T., Yang J.C., Yang M.F. (2022). Environmental Toxic Metal Contaminants and Risk of Stroke: A Systematic Review and Meta-Analysis. Environ. Sci. Pollut. Res. Int..

[69] Mao L., Lu J., Wen X., Song Z., Sun C., Zhao Y., Huang F., Chen S., Jiang D., Che W. (2025). Cuproptosis: Mechanisms and Nanotherapeutic Strategies in Cancer and Beyond. Chem. Soc. Rev..

[70] Chen X., Cai Q., Liang R., Zhang D., Liu X., Zhang M., Xiong Y., Xu M., Liu Q., Li P. (2023). Copper Homeostasis and Copper-Induced Cell Death in the Pathogenesis of Cardiovascular Disease and Therapeutic Strategies. Cell Death Dis..

[71] Zhu Z., Song M., Ren J., Liang L., Mao G., Chen M. (2024). Copper Homeostasis and Cuproptosis in Central Nervous System Diseases. Cell Death Dis..

[72] Chen Z., Liu X., Wu Y., Qi X., Ling Q., Wu Z., Shi Y., Hu H., Yu P., Ma J. (2024). Association between Serum Copper Levels and Stroke in the General Population: A Nationally Representative Study. J. Stroke Cerebrovasc. Dis..

[73] Storves K.P., Talcott M.R., Wallace J.M., Bennett B.T., Makaron L.M., Clemons D., Hugh (Chip) Price V., Cohen J.K., Hasenau J.J., Freed C.K. (2025). The Veterinary Consortium for Research Animal Care and Welfare Survey on Revisions to the Eighth Edition of the Guide for the Care and Use of Laboratory Animals. J. Am. Assoc. Lab. Anim. Sci..

[74] du Sert N.P., Hurst V., Ahluwalia A., Alam S., Avey M.T., Baker M., Browne W.J., Clark A., Cuthill I.C., Dirnagl U. (2020). The ARRIVE Guidelines 2.0: Updated Guidelines for Reporting Animal Research. PLoS Biol..

[75] Pomierny B., Krzyżanowska W., Skórkowska A., Jurczyk J., Budziszewska B., Pera J. (2024). Chicago Sky Blue 6B Exerts Neuroprotective and Anti-Inflammatory Effects on Focal Cerebral Ischemia. Biomed. Pharmacother..

[76] Ali M.H.M., Rakib F., Abdelalim E.M., Limbeck A., Mall R., Ullah E., Mesaeli N., McNaughton D., Ahmed T., Al-Saad K. (2018). Fourier-Transform Infrared Imaging Spectroscopy and Laser Ablation -ICPMS New Vistas for Biochemical Analyses of Ischemic Stroke in Rat Brain. Front. Neurosci..

