# Supplementary material for: Metallomic Aspects of Stroke and Recovery: ICP-MS Study with Chemometric Analysis
Source: Molecules. 2025 Dec 5;30(24):4672. doi: 10.3390/molecules30244672 (PMC12736056; doi:10.3390/molecules30244672)
Supplement: Supplementary file 1 [file molecules-30-04672-s001.zip › molecules-3995206-supplementary.pdf]

Table S1. The range of concentration of elements and the observed statistical significances.

| Element | Range of concentration mean values (± SD) | Unit | Observed statistical significances |
|---|---|---|---|
| Mg | 160.4±12.7 – 247±17.1 | ppm | CC: *p<0.05 vs SHAM<br>HIPP: ####p<0.0001 vs SHAM |
| Na | 1053±104.9 – 1334±99.7 | ppm | HIPP: ##p<0.01 vs SHAM |
| K | 3346±111.1 – 3892±225.2 | ppm | CC: **p<0.01 vs SHAM<br>HIPP: #p<0.05 vs SHAM |
| P | 2604±130.6 – 3224±216.2 | ppm | CC: *p<0.05 vs SHAM<br>HIPP: ##p<0.01 vs SHAM |
| Ca | 148.4±6.5 – 437.9±39.8 | ppm | CC: ****p<0.0001 vs SHAM<br>CDS:^^^^p<0.0001vsSHAM HIPP:<br>####p<0.0001 vs SHAM |
| Cu | 2.5±0.17 – 28.1±1.7 | ppm | CC: ****p<0.0001 vs SHAM<br>CDS:^^^^p<0.0001 vs SHAM |
| Zn | 14.6±0.4 – 24±2 | ppm | CC: ***p<0.001 vs SHAM<br>HIPP: #p<0.05 vs SHAM |
| Fe | 9.9±1.8 – 22.7±2.7 | ppm | CC: ****p<0.0001 vs SHAM<br>CDS:^^^^p<0.0001 vs SHAM |
| Cr | 60.8±10 – 240.2±30.6 | ppb | CC: ***p<0.001 vs SHAM HIPP:<br>##p<0.01 vs SHAM |
| Mn | 240.5±16.5 – 442±27 | ppb | CC: ****p<0.0001 vs SHAM<br>HIPP: ####p<0.0001 vs SHAM |
| V | 25.9±5.4 – 64.1±9 | ppb | CC: ***p<0.001 vs SHAM<br>CDS:^^^p<0.0001 vs SHAM |
| Se | 141.3±39.2 – 341.4±42.2 | ppb | CDS:^^p<0.01 vs SHAM<br>HIPP: ###p<0.001 vs SHAM |
| B | 255.5±76 – 1328±54.4 | ppb | CC: ***p<0.001 vs SHAM<br>HIPP: ####p<0.0001 vs SHAM |
| Al | 3140±457.2 – 6975±693 | ppb | CC: ***p<0.001 vs SHAM<br>HIPP: ####p<0.0001 vs SHAM |
| Pb | 27.6±2.9 – 466.2±55 | ppb | CDS:^^^^p<0.0001 vs SHAM<br>HIPP: ####p<0.0001 vs SHAM |
